# Usefulness of Cerebrospinal Fluid Alzheimer's disease biomarkers in older patients: Evidence from a national multicenter prospective study

**DOI:** 10.1016/j.tjpad.2024.100009

**Published:** 2025-01-01

**Authors:** Théodore Decaix, François Mouton-Liger, Julien Dumurgier, Emmanuel Cognat, Agathe Vrillon, Jacques Hugon, Claire Hourregue, Elodie Bouaziz-Amar, David Wallon, Muriel Quillard Muraine, Anne-Cécile Troussière, Eloi Magnin, Emmanuelle Duron, Nathalie Philippi, Frédéric Blanc, Audrey Gabelle, Bernard Croisile, Alain Jager, Florence Pasquier, Susanna Schraen, Vincent de la Sayette, Émilie Beaufils, Carole Miguet-Alfonsi, Claire Paquet, Matthieu Lilamand

**Affiliations:** a Geriatrics Department, Fernand Widal Lariboisière University Hospital, GHU APHP.Nord, Paris, France; b Paris-Cité University, CNRS, CitCoM, F-75006, Paris, France; c Paris-Cité University, Inserm U1144, Paris, France; d Cognitive Neurology Center, Fernand Widal Lariboisière University Hospital, GHU APHP.Nord, Paris, France; e Paris-Cité University, Inserm U1153, Paris, France; f Biochemistry Department, Fernand Widal Lariboisière University Hospital, GHU APHP.Nord, Paris, France; g Univ Rouen Normandie, Normandie Univ, Inserm, U1245; h CHU Rouen, Department of Neurology and CNRMAJ, F-7600, Rouen, France; i Department of General Biochemistry, CHU Rouen, F-76000 Rouen, France; j Consultation mémoire, centre hospitalier de Versailles, 177 rue de Versailles, 78150, Le Chesnay; k Université de Franche-Comté, UMR INSERM 1322 LINC, CMRR, service de neurologie, CHU Besançon, F-25000 Besançon; l Service Hospitalo-Universitaire de gériatrie. Assistance Publique-Hôpitaux de Paris, Hôpitaux Universitaires Paris-Saclay, Hôpital Paul-Brousse Villejuif FR, 12 Avenue Paul Vaillant Couturier, Villejuif, 94800, France; m CESP, Team MOODS, Université Paris-Saclay, UVSQ, Le Kremlin-Bicêtre, France; n CMRR (Memory Resources and Research Centre), Neurology Service and Geriatrics Department, University Hospital of Strasbourg, Strasbourg, France; o ICube Laboratory UMR 7357, University of Strasbourg and CNRS, Strasbourg, France; p Memory Research and Resources Center, Department of Neurology, Chu Gui de Chauliac, University of Montpellier, Institute of Neurosciences of Montpellier, France; q Service de neuropsychologie, Hospices Civils de Lyon, Hôpital Neurologique Pierre Wertheimer, 69677 Bron Cedex, France; r Cabinet de neurologie SCM Jager-Gal, 6 place du Luxembourg, 57100, Thionville, France; s Univ. Lille, Inserm, CHU Lille, UMR‐S1172, LiCEND, Lille Neuroscience & Cognition, LabEx DISTALZ, Lille, France; t Service de neurologie, CHU de Caen, France; u Centre Mémoire Ressources et Recherche (CMRR), Centre Hospitalier Universitaire de Tours, Tours, France; v Service de biochimie, Hôpital Universitaire de Besançon, 2500, Besançon, France

**Keywords:** Clinical practice, Cognitive impairment, Memory clinics, Amyloid-beta, Tau protein

## Abstract

**Background:**

The use of cerebrospinal (CSF) biomarkers in the diagnosis of Alzheimer's disease (AD) has been gaining interest in clinical practice. Although their usefulness has been demonstrated, their potential value in older patients remains debated.

**Objectives:**

To assess whether knowledge of the results of CSF AD biomarkers was associated with the same gain in diagnostic confidence in older adults > 80 than in younger patients.

**Design:**

Prospective multicenter study, including memory clinics physicians who completed a two-part questionnaire for all their patients addressing the requirement for assessment of Alzheimer's disease biomarkers in CSF proposed as part of routine care during the study period.

**Setting:**

30 secondary or tertiary memory clinics in France.

**Measurements:**

Clinicians indicated their diagnosis hypothesis and an estimate of their diagnostic confidence [scale 1–10]. Receiver operating characteristic (ROC) analysis, including the calculation of the area under the curve (AUC), was conducted using logistic regression to evaluate the diagnostic performance of CSF AD biomarkers.

**Results:**

In 813 consecutive patients, median age 70 [interquartile range (IQR) = 63 – 77] including 132 patients over 80 years, we observed a similar confidence gain in CSF biomarkers between older and younger patients, both for AD and non-AD diagnoses. In older patients, the added value of CSF biomarkers was greater when CSF biomarkers indicated AD profile whereas the initial hypothesis was “non-AD”, leading to a final diagnosis of AD (2.4 ± 1.6 versus 1.1 ± 2.1, p-value, *p* = 0.03). ROC analyses showed similar performance of AD CSF biomarkers in older and younger patients.

**Conclusion:**

CSF AD biomarkers added substantial value to clinical assessment in patients over 80. Their use seems crucial in the diagnostic process for older adults referred to memory clinics.

## 1 Introduction

Over the last two decades, the increasing adoption of Alzheimer's disease (AD) biomarkers, such as cerebrospinal fluid (CSF) β-amyloid and tau, as well as nuclear medicine biomarkers indicating amyloid and tau deposition, has improved clinicians’ diagnostic accuracy in regular practice [[1], [2], [3], [4]]. These tools allow the in vivo identification of core lesions in AD and, thus, earlier diagnosis. They have also proven valuable for clinicians in assessing and differentiating various neurocognitive disorders [2]. Thus, the diagnosis of clinical AD without biomarker assessment was associated with 20–30% of misdiagnoses, according to post-mortem neuropathological analyses [5,6]. This breakthrough in diagnostic methodologies has the potential to improve our understanding and management of neurodegenerative conditions, ultimately leading to more effective therapeutic interventions [7]. Accordingly, the amyloid and tau biomarkers were incorporated into the last revised definitions of AD for research purpose [[8], [9], [10]].

The use of these CSF biomarkers has been suggested as reliable in diagnosing neurodegenerative disease in older patients as in diagnosing younger patients [11]. Low levels of peptide Aß42, combined with an increase in phosphorylated Tau protein in the CSF in older patients, can robustly predict the risk of cognitive decline and the onset of dementia [[12], [13], [14], [15]]. However, several controversies specific to older patients have called the implementation of these tools in clinical geriatric medicine and among older patients in memory clinics into question [16]. Deciphering CSF biomarkers in older patients is challenging because of the prevalence of cerebral damage resulting from vascular issues or other neurodegenerative causes [[17], [18], [19]]. Thus, older patients may present amyloid biomarker positivity independent from AD associated with another cause of cognitive impairment. Besides, very few cognitive impairment situations are due to a single etiological mechanism. In addition, CSF is collected via lumbar puncture (LP), a procedure often considered by clinicians to be difficult to perform in older patients. Older patients may present with lumbar osteoarthritis, and LP can be associated with side effects (pain and anxiety) and potential complications [20,21]. However, post-LP complications are uncommon and mostly benign; their incidence decreases with age [22,23]. In France, geriatricians working in secondary and tertiary memory centers are qualified to perform LP for CSF biomarker testing, but their ability depends on their experience in clinical practice.

In a qualitative study, healthcare professionals in geriatrics also showed reluctance to perform LP in older patients, depending on their own experiences regarding LP and CSF biomarkers [24]. For these reasons, clinicians’ interest in knowing the results of AD biomarkers in older patients remains unclear and varies depending on the context of their practice [25]. A previous study assessed the influence of clinicians’ awareness of CSF biomarker results on their diagnostic approach in memory clinics [26]. Clinicians were likely to trust the results of AD CSF biomarkers in terms of confirming their initial diagnostic hypothesis or changing their diagnostic hypothesis when faced with inconsistent biomarker results. The main objective of the present clinical study was to determine whether knowledge of the results regarding CSF AD biomarkers was associated with the same increase in diagnostic confidence regarding patients over 80 as compared to younger patients. We also investigated clinicians’ attitudes when faced with inconsistent biomarker results compared with their initial diagnostic hypothesis.

## 2 Methods

### 2.1 Study population and design

This study was designed to investigate the potential impact of CSF biomarker results on the diagnostic hypothesis made by experienced clinicians in their daily routine approach. This was a nationwide observational, prospective, multicenter study conducted in France between February 2012 and July 2019 in 30 recruitment centers (secondary or tertiary memory clinics).

Participation in the study was offered to all clinicians practicing in these centers, regardless of their experience with the CSF AD biomarkers. All centers employed comparable clinical and biochemical procedures for the purpose of diagnosis, as well as internationally validated criteria for AD [3,27]. All patients underwent a thorough clinical and neuropsychological examination and brain imaging. In this study, when clinicians considered a patient eligible for CSF biomarkers, they filled an anonymized patient questionnaire (**See details in supplementary material**) [22].

CSF AD biomarkers assessment was performed in 18 local laboratories, according to the geographical source of the samples. All biochemistry teams participated in two national cohorts and shared the same standards for CSF analysis and interpretation [[28], [29], [30], [31], [32]]. In addition, two external quality controls were performed by the involved laboratories: one by a working group of the *Société Française de Biologie Clinique* and the other by the Alzheimer's Association [33]. All centers used the same tubes for CSF collection (Sarstedt ref 62.610.201) and similar approaches for measuring the core AD biomarkers in CSF. CSF profiles were classified according to the AT(N) classification, using thresholds established by each laboratory based on their own population data and/or the manufacturers' recommendations. The same cut-offs were applied to define the AT(N) classification for all participants. Based on the CSF biomarker results, three possible interpretations were made according to the AT(N) profile: "in favor of AD" (typically an *A* + *T*+ profile with low CSF Aβ42 level and or decreased Aβ42/Aβ40 ratio associated with elevated CSF P-tau and elevated CSF t-tau levels), "non in favor of AD" (typically an A- T- profile) and "non-contributory" (CSF with discordant biomarkers such as A- *T* + *N*- or results close to thresholds) (See details in supplementary material). If no interpretation was provided, the CSF biomarker results were categorized as "without interpretation”. Some lumbar punctures were failed or encountered technical problems (e.g. hemorrhagic CSF) and were then referred to as "with technical problems". The clinicians were then asked again to indicate their final diagnostic hypothesis with knowledge of CSF biomarkers.

### 2.2 Variables of interest

The study population was divided into 2 groups: older and younger patients. Patients were allocated to the older patient's group if their age was ≥ 80 and to the younger patient's group otherwise.

The initial and/or final diagnosis was AD if the diagnosis given by the clinician was “AD”, “MCI due to AD” or “primary progressive aphasia due to AD”. Otherwise, the diagnosis was considered non-AD. The initial diagnosis corresponded to the diagnostic hypothesis with the higher likelihood established blind to the results of the CSF biomarkers, and the final diagnosis corresponded to that after the detection of the CSF biomarkers.

Changed diagnosis corresponded to cases in which the initial diagnosis was AD and the final diagnosis was non-AD, and vice versa. Unchanged diagnosis corresponded to cases in which the initial and final diagnoses were consistent. The change in confidence based on the visual numerical scale was calculated (change in confidence).

### 2.3 Ethics

Due to the utilization of anonymized observational data provided by clinicians in this study, the requirement for Ethics Committee approval was waived. Therefore, informed consent from the patients was not required. Nevertheless, we obtained the required approval from the French *Commission Nationale Informatique et Liberté* to collect anonymized data from these patients.

### 2.4 Statistical analysis

All statistical tests were performed to compare the older and younger patients based on the aforementioned parameters. Student's *t*-test or Mann–Whitney U test was used to compare continuous variables depending on the normal distribution of variables. Chi-square tests or Fisher's exact test were used to compare categorical variables. The Hodges Lehman (HL) estimator was used to quantify the observed differences in delta confidence between older and younger patients [34]. The main analysis excluded CSF biomarkers “non-contributory”, “with technical problems” and “without interpretation”. Additional analyses were performed based on CSF biomarkers “non-contributory”. Receiver operating characteristic (ROC) analysis, including the calculation of the area under the curve (AUC), was conducted using logistic regression to evaluate the diagnostic performance of CSF AD biomarkers. P-value < 0.05 was considered statistically significant. All analyses were performed using R 4.1.2 software.

## 3 Results

**Fig. 1:**
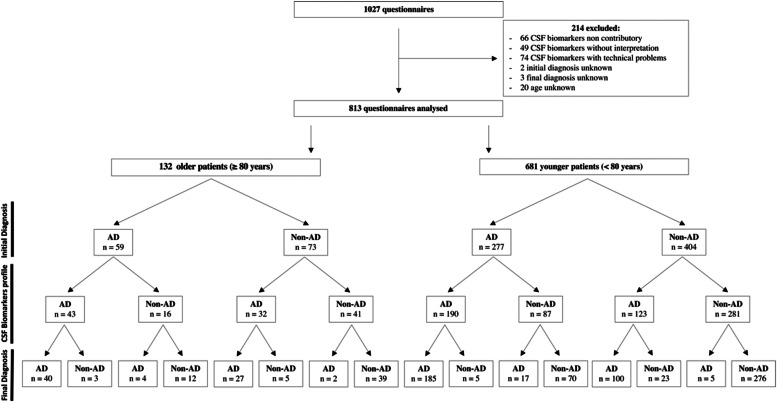
Study Flowchart. AD, Alzheimer's Disease; CSF, cerebrospinal fluid; Non-AD, non-Alzheimer's Disease.

The study flowchart is shown in Fig. 1. From the 1027 questionnaires collected during the study period, 214 questionnaires were excluded from the main analysis due to CSF biomarkers was non-contributory (*n* = 66), without interpretation (*n* = 49), with technical problems (*n* = 74) or when the initial diagnosis (*n* = 2), final diagnosis (*n* = 3), or age (*n* = 20) were not indicated in the questionnaire. Fourteen older and 52 younger patients had CSF biomarkers classified as non-contributory (*p* = 0.4) (**data not shown**). A total of 813 questionnaires were analyzed, with 132 corresponding to older patients and 681 to younger ones. The mean time from initial to final diagnosis was 51.1 (standard deviation = 32.4) days.

The characteristics of the study population are presented in Table 1. The overall median age was 70 [interquartile range (IQR) = 63 – 77]. In older and younger patients, median age was 83 (81 – 84) and 67 (61 – 73), respectively (*p* < 0.0001). There were no significant differences between the older and younger patients in terms of initial diagnosis of AD and non-AD (*p* = 0.45) with a higher level of confidence in younger patients (*p* = 0.046). The proportion of AD biological profile was higher in older than in younger patients (56.8% versus 46.0%, *p* = 0.03). The proportion of AD and non-AD patients differed for the final diagnosis compared with the initial diagnosis, with 55% AD in older patients and 45.1% in younger patients (*p* = 0.04), with no significant difference in the level of confidence or in the percentage of changed diagnosis (33.3% in older versus 26.4% in younger patients, *p* = 0.10). By comparing non-contributory CSF biomarkers (*n* = 66) to the overall sample (*n* = 813), there were more initial and final diagnosis of AD (54.4% versus 41.3%, *p* = 0.0001 and 65.2% versus 46.7%, *p* = 0.006, respectively) with a smaller percentage of changed diagnosis (24.2% versus 27.5%, *p* = 0.03) (**data not shown**). Anticholinesterase inhibitors (ChEIs) were introduced in 34.8% of older patients and 30.0% of younger patients (*p* = 0.048). The final diagnosis associated with prescription of ChEIs was AD in 93.5% and 92.6% of older and younger patients, respectively.

**Table 1:** Characteristics of the study population.

	Older (≥ 80 years)	Younger (< 80 years)	*p*	Overall
**N (%)**	**132**	**681**		**813**
Age (years), median (IQR)	83 (81 – 84)	67 (61 – 73)	< 0.0001	70 (63 – 77)
Male sex, *n* (%)	61 (46.2)	337 (49.5)	0.56	398 (48.9)
Initial diagnosis				
AD, *n* (%)	59 (44.7)	277 (40.7)	0.45	336 (41.3)
Non-AD, *n* (%)	73 (55.3)	404 (59.3)	_	477 (58.6)
Confidence level, median (IQR)	6 (5 - 7)	6 (6 - 7)	0.046	6 (6 – 7)
CSF biomarkers in favor of AD, *n* (%)	75 (56.8)	313 (46.0)	0.03	388 (47.7)
Final diagnosis				
AD, *n* (%)	73 (55.3)	307 (45.1)	0.04	380 (46.7)
Non-AD, *n* (%)	59 (44.7)	374 (54.9)	_	433 (53.3)
Confidence level, median (IQR)	8 (7 - 9)	8 (7 - 9)	0.69	8 (7 – 9)
Changed diagnosis, *n* (%)	44 (33.3)	180 (26.4)	0.13	224 (27.5)
ChEIs management				
ChEIs introduction, *n* (%)	46 (34.8)	177 (30.0)	0.048	223 (27.4)
ChEIs maintenance, *n* (%)	1 (0.7)	6 (0.9)	1.0	7 (0.9)
ChEIs stop, *n* (%)	7 (5.3)	22 (3.2)	0.30	29 (3.6)

The data presented as mean ± standard deviation, median (interquartile range), and number (percentage). A p-value < 0.05 is considered significant. Missing data: male sex (*n* = 11), confidence level (*n* = 1).

AD, Alzheimer's Disease; CSF, cerebrospinal fluid; ChEIs, cholinesterase inhibitors; NA, missing data; Non-AD, non-Alzheimer's Disease.

**Fig. 2:**
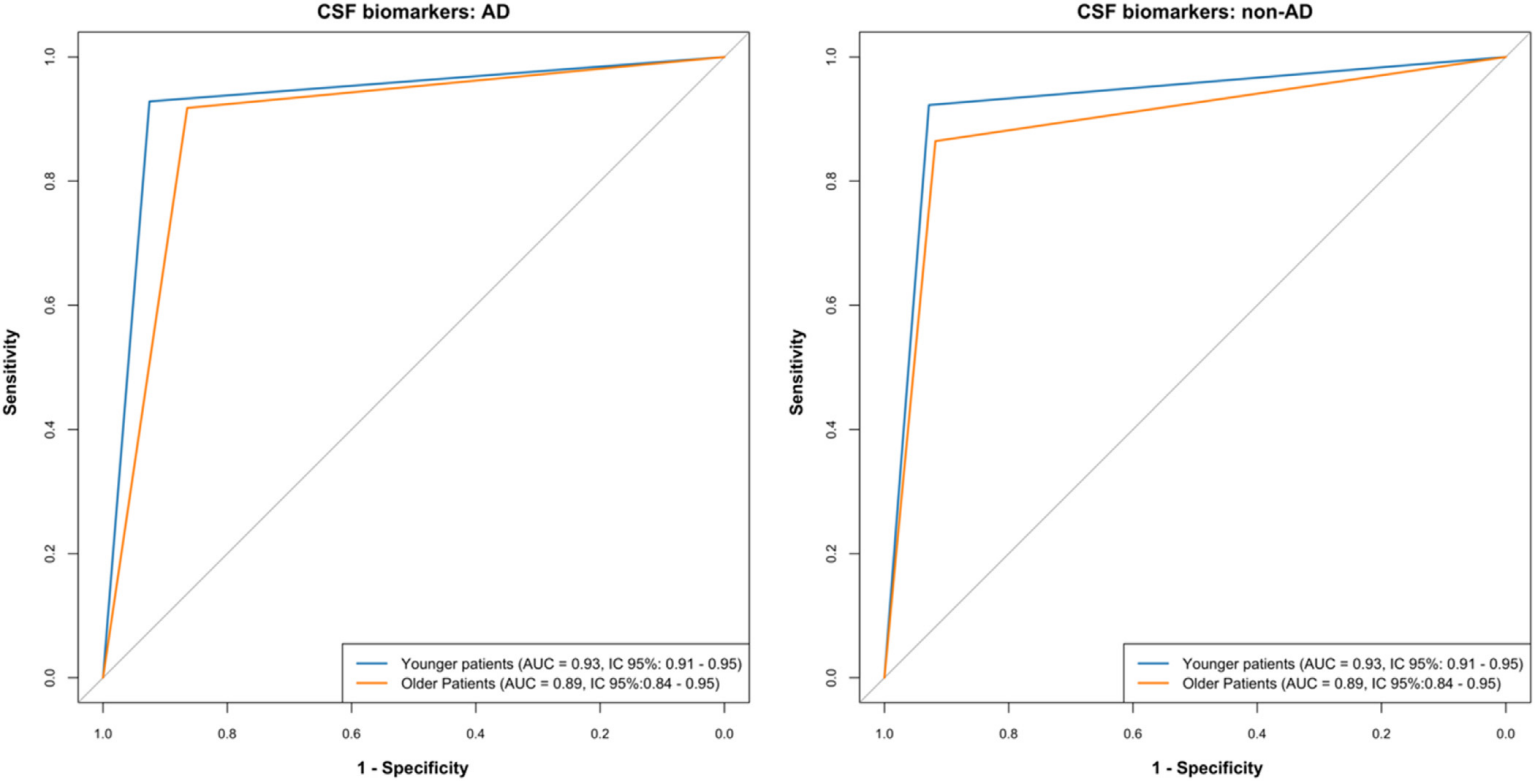
Performance of Alzheimer's Disease cerebrospinal fluid biomarkers (ROC analysis). AD, Alzheimer's Disease; AUC, Area Under the Curve; CSF, cerebrospinal fluid; Non-AD, non-Alzheimer's Disease.

Among 16 older and 87 younger patients with an initial diagnosis of AD and CSF biomarkers non-AD, 12 older (75.0%) and 70 younger patients (80.5%) had their final diagnosis changed to non-AD (*p* = 0.73). In the case of a mismatch with an initial diagnosis of non-AD and CSF biomarkers in favor of AD, 27 of 32 older (84.4%) and 100 of 123 younger (81.1%) patients had their final diagnosis changed to AD (*p* = 0.80). The non-AD diagnosis was unchanged in 15.6% of older patients and 18.7% of younger patients, despite CSF biomarkers in favor of AD. Among the 43 older patients with an initial diagnosis of AD and CSF biomarkers in favor of AD, the diagnosis was unchanged in 40 patients (93%); as well as in 185 out of 190 (97.4%) younger patients (*p* = 0.17). The diagnosis of non-AD was also unchanged in important and similar proportions after the results of the CSF biomarkers non-AD in 95.1% and 98.2% of older and younger patients, respectively (*p* = 0.22). CSF biomarkers in favor of AD provided a diagnostic performance of the same magnitude in older and younger patients for the confirmation of AD diagnosis (AUC = 0.89, IC95% 0.84 – 0.95 versus AUC = 0.93, IC95% 0.91 – 0.95, respectively) (Fig. 2). The same trend was observed in the case of CSF biomarkers non in favour of AD to rule out the diagnosis of AD (AUC = 0.89, IC95% 0.84 – 0.95 in older patients versus AUC = 0.93, IC95% 0.91 – 0.95 in younger patients). The addition of non-contributory CSF biomarkers in the ROC analysis had minimal impact on test performance, both in the case of CSF biomarkers in favor of AD and those not in favor of AD (AUC = 0.85, IC95% 0.80 – 0.91 in older versus AUC = 0.92, IC95% 0.90 – 0.94 in younger patients and AUC = 0.85, IC95% 0.80 – 0.91 in older versus AUC = 0.92, IC95% 0.90 – 0.94 in younger patients, respectively) (**data not shown**).

The clinicians' level of confidence before and after the results of the CSF biomarkers is reported in Table 2. The change in confidence was greater in older patients than in younger ones in the scenario: initial diagnosis = non-AD - CSF biomarkers = AD – final diagnosis = AD [2.4 (1.6) vs 1.0 (2.1) respectively (*p* = 0.03)]. This difference was important 1.0 (95% CI 9.3e-06 – 2.0) according to the HL estimator. In the case of initial diagnosis AD and final diagnosis non-AD, this change in confidence was also higher in older with non-contributory CSF-biomarkers [1.4 (1.8) vs −0.7 (0.8), *p* = 0.03] (**data not shown**). In cases of concordance between the initial diagnosis and the CSF biomarkers, the diagnosis was unchanged with a change in confidence of 2.1 (1.4) in older patients and 2.1 (1.2) in younger patients for AD (*p* = 0.99) and 2.0 (1.6) in older patients and 1.5 (1.5) in younger patients for non-AD (*p* = 0.10). When the initial diagnosis was AD and the CSF biomarkers were non-AD, the change in confidence in the change of diagnosis for non-AD was 1.9 (2.0) in older and 1.8 (1.4) in younger patients (*p* = 0.70).

**Table 2:** Comparison of the change in confidence between older and younger patients according to CSF biomarkers for establishing the final diagnosis.

Initial diagnosis	CSF Biomarkers	Final Diagnosis	Older	Younger	*p*	HL estimator (95% CI)
AD	AD	AD	2.1 ± 1.4	2.1 ± 1.2	0.99	2.9e-04 (4.9e-05 – 5.8e-05)
Non-AD	Non-AD	Non-AD	2.0 ± 1.6	1.5 ± 1.5	0.10	5.5e-05 (−6.3e-05 – 0.99)
Non-AD	AD	AD	2.4 ± 1.6	1.0 ± 2.1	0.03	1.0 (9.3e-06 – 2.0)
AD	Non-AD	Non-AD	1.9 ± 2.0	1.8 ± 1.4	0.70	6.3e-05 (−0.99 – 0.99)

The data presented as mean ± standard deviation. A p-value < 0.05 is considered significant.

AD, Alzheimer's Disease; CSF, cerebrospinal fluid; HL, Hodges-Lehmann estimator; Non-AD, non-Alzheimer's Disease.

## 4 Discussion

In this real-life, multicenter, prospective study, the relationship between the awareness of CSF biomarker results and diagnostic confidence among clinicians in French memory clinics was investigated, with a particular emphasis on older adults. Clinicians showed a high level of confidence in CSF biomarkers, as final diagnoses were in line with the CSF biomarkers in 89.4% of older patients and 92.6% of younger patients [26]. To the best of our knowledge, this is the first analysis highlighting the usefulness of these diagnostic tools in clinical practice in patients over 80. In the most common case of concordance between the initial diagnostic hypothesis and the CSF biomarker results, the diagnosis was unchanged, with a similar increase in confidence in both older and younger patients. When the hypothesis and the CSF biomarkers were mismatched and the initial diagnosis was non-AD, clinicians were likely to change their diagnosis with a greater level of confidence in older than in younger patients. Overall, the chances of maintaining or changing in the initial diagnosis based on the CSF biomarkers did not differ between the two age groups. Furthermore, in all cases of the suspicion/non-suspicion of AD and CSF biomarkers that were in favor/not in favor of AD, the level of confidence in CSF biomarker results knowledge was increased by approximately two points on a visual numerical scale for both groups.

Several factors may explain the greater gain in confidence regarding older patients when the initial hypothesis was non-AD but the CSF biomarkers were in favor of AD. The clinical presentation of dementia may be less specific in older patients as compared to younger ones because of the presence of confounding factors. For example, visual impairment, hearing loss, mood disorders, sleep problems and anticholinergic treatments are common in this population and may impact the results of neuropsychological tests [[35], [36], [37], [38], [39], [40]]. Symptoms such as dysautonomia and parkinsonism are particularly prevalent in older patients and may mislead clinicians into incorrectly diagnosing such patients as having dementia with Lewy bodies (DLB) or tauopathies [[41], [42], [43]]. Moreover, in cognitively impaired older patients, an overlap in symptomatology exists between vascular cognitive impairment and AD, which complicates making a clear diagnosis [[44], [45], [46]]. Significant proportions of individuals with co-pathologies linked to DLB, argyrophilic grains or limbic-predominant age-related TDP-43 encephalopathy (LATE) along with AD are susceptible to a heightened risk of accelerated cognitive decline [47]. Thus, low levels of CSF Aß42 are associated with faster cognitive decline in patients with DLB or LATE [48,49]. Therefore, it is difficult to make a precise diagnosis in older patients based solely on neuropsychological assessment and brain-imaging data. Furthermore, in our study, regardless of the initial diagnosis, the level of confidence appears to be lower in older, as compared to younger, patients before the results regarding CSF biomarkers are known. Therefore, CSF biomarkers enhance clinicians’ confidence in diagnosing AD by emphasizing the presence of core pathophysiological lesions, particularly given challenging or atypical presentations.

Both CSF biomarkers and clinicians’ final diagnoses were more often in favor of AD in older patients as compared to younger patients in our study. Advanced age is an established risk factor for AD, and clinicians were likely to suspect this diagnosis in most cases of dementia with memory loss. The prevalence of frontotemporal dementia is lower in older adults [50]. LATE is frequent in this population, but its diagnosis is based on neuropathological characteristics, which cannot yet be demonstrated in vivo [49]. The involvement of vascular lesions or brain co-pathologies in cognitive impairment is challenging [51,52]. Therefore, given co-pathologies, a diagnosis of AD may often be prioritized over others as one of the most plausible explanations for cognitive impairment in complex situations. If AD is suspected after a neuropsychological assessment, only amyloid- and tau-negative CSF biomarker results can rule out AD with a high negative predictive value [53]. Consequently, the use of CSF biomarkers may avoid false diagnoses of AD in this population.

To our knowledge, there is no real-life study collecting the diagnostic attitude of memory center clinicians according to the results of CSF AD biomarkers in older adults. However, studies have investigated the reproducibility of CSF AD biomarkers in real life and highlighted the contribution of the Aß40/Aß42 ratio to diagnosis [54,55]. In our study, most patients classified as *A*+ had undergone an Aß40/Aß42 ratio assay. Another study reported on the usefulness of CSF AD biomarkers in routine clinical practice for atypical clinical presentations [56]. The older population covered by our study is particularly concerned by this type of presentation. Finally, Rosenberg et al. assessed eligibility for therapies anti-amyloid based on real-life CSF AD biomaker assays [57].

The emergence of anti-amyloid therapies for AD highlights the critical need for biomarker-based diagnosis to access to these disease-modifying treatments. The CLARITY AD study included patients up to 90 years old; upon further analysis and subgroup examination, it was demonstrated that older patients could experience similar benefits from this treatment as compared to their younger counterparts [58,59]. Therefore, the use of AD biomarkers measurement is likely to increase in the coming years in both older and younger patients [25]. However, their use in the older population must be carefully evaluated, especially in the case of multimorbid patients, who would be denied anti-amyloid therapies according to their overall poor functional status. Thus far, the decision to measure CSF biomarkers in older patients appears to be influenced more by clinicians’ habits or beliefs, rather than being driven by evidence-based medicine [24]. A comprehensive geriatric assessment, which evaluates the patient's physiological age, as well as the patient's willingness to understand their diagnosis, may provide guidance on the appropriate use of CSF biomarkers in older adults.

This study has certain limitations. (1) Only three patients over 90 years old were included in this study, indicating a barrier to the use of CSF biomarkers in the oldest patients or a selection bias in memory clinics. (2) The data were collected between 2012 and 2019, which does not accurately reflect clinicians’ attitudes towards CSF biomarkers in 2024. (3) Extensive data regarding clinician characteristics (e.g., years of experience and medical specialty) were lacking. (4) As we only included patients who underwent LP for biomarker assessment, we cannot exclude a selection bias regarding the clinical presentation of these individuals. For example, biomarker measurement may have been less performed in older adults with typical clinical AD, increasing the proportion of atypical AD presentation in our study population. (5) Some CSF biomarkers were excluded from the main analysis, as our aim was to assess the impact of a “positive” or “negative” result on the clinician's initial diagnosis, which could lead to an overestimation of test performance. However, considering non-contributory CSFs showed a weak impact on test accuracy.

## 5 Conclusion

CSF AD biomarkers have led to major improvements in clinical practice in terms of early and accurate diagnoses. This study demonstrated that similar benefits were associated with their use in both older and younger patients. In addition, they seem to be a powerful tool for use in ruling out AD in the absence of brain amyloid and tau deposition. These biomarkers will likely attract additional further interest with the advent of new immunotherapies and their application in older patients. Further research is needed to complete this work and define the optimal framework for use in employing AD biomarkers among the oldest people.

## Funding

None.

## Declaration of competing interest

On behalf of all authors, the corresponding author states that there is no conflict of interest.

## Appendix Supplementary materials

Image, application 1

Image, application 2

Image, application 3
