# Supplementary material for: Usefulness of Cerebrospinal Fluid Alzheimer's disease biomarkers in older patients: Evidence from a national multicenter prospective study
Source: J Prev Alzheimers Dis. 2025 Jan 1;12(1):100009. doi: 10.1016/j.tjpad.2024.100009 (PMC12184015; doi:10.1016/j.tjpad.2024.100009)
Supplement: Supplementary file 1 [file mmc1.docx]

**Supplementary material: Usefulness of Cerebrospinal Fluid Alzheimer’s disease biomarkers in older patients: evidence from a national multicenter prospective study.**

**Study questionnaire**

This questionnaire was created by a team of neurologists and biologists and was validated in a previous study.

*First part*

The first part was completed by the clinician before CSF biomarker assessment, typically on the day of the LP procedure or when the patient was referred to a day hospital for CSF biomarkers assessment. Clinicians were asked to indicate the patient’s age and sex as well as the examinations performed before LP (neuropsychological tests, brain MRI, FDG-PET, DOPA-PET, or other). Finally, the clinicians formulated their diagnostic hypothesis with increased confidence: AD, mild cognitive impairment (MCI), frontotemporal dementia, vascular dementia, semantic dementia, primary progressive aphasia, Parkinson’s disease, Lewy body disease, progressive supranuclear palsy, Creutzfeldt-Jacob disease, toxic encephalopathy, psychiatric disorder, or other). They were asked to indicate their confidence in this diagnosis using the same visual numerical scale ranging from 1 to 10. The completion of the second part occurred after the performance of the LP, as the results of the CSF AD biomarkers were available.

*Second part*

As in the first part, they were asked to indicate their confidence in this diagnosis using a visual numerical scale ranging from 1 to 10. At the end of this second part, the clinicians also reported on therapeutic management (no change in treatment, introduction/maintenance/stop of anticholinesterase/anti-NMDA treatment, or other) and medico-social management (no change, introduction of financial aid, inclusion in another study, or other).
