# Supplementary material for: Usefulness of Cerebrospinal Fluid Alzheimer's disease biomarkers in older patients: Evidence from a national multicenter prospective study
Source: J Prev Alzheimers Dis. 2025 Jan 1;12(1):100009. doi: 10.1016/j.tjpad.2024.100009 (PMC12184015; doi:10.1016/j.tjpad.2024.100009)
Supplement: Supplementary file 3 [file mmc3.pdf]

Impact clinique du dosage des biomarqueurs du liquide cérébro-rachidien (LCR) dans la prise en charge des maladies neurodégénératives.

Patient. ☐ Homme ☐ Femme Année de naissance (YYYY) : _ _ _ _

LE JOUR DE LA DEMANDE DE BIOMARQUEURS

Date : _ _ /_ _ / _ _ _ _

1. La demande actuelle se fait lors :

☐ De la première consultation ☐ De la deuxième consultation ☐ > 2ème consultation
☐ > 3 mois de suivi ☐ > 6 mois de suivi ☐ > 1 an de suivi

2. Le patient a t-il déjà eu d'autres examens complémentaires avant la demande de ponction lombaire?

☐ IRM/ TDM cérébrale ☐ PET/ scintigraphie cérébrale ☐ Autre ponction lombaire (PL)
☐ Bilan neuropsychologique ☐ Bilan orthophonique ☐ Autres (préciser) :

Si ces examens complémentaires sont prescrits en même temps que la PL, ne pas cocher ces cases.

3. Quelle est votre (vos) hypothèse(s) diagnostique(s) le jour où vous demandez la PL ?[3]

☐ Maladie d'Alzheimer (MA) ☐ MCI ☐ Démence fronto-temporale
☐ Démence vasculaire ☐ Démence sémantique ☐ Aphasie progressive (APL/APNF[4])
☐ Démence parkinsonienne ☐ Cause toxique (alcool) ☐ Maladie à prions
☐ Dégénérescence cortico-basale ☐ Paralysie supranucléaire ☐ Démence à corps de Lewy
☐ Troubles psychiatriques ☐ Atrophie focale progressive ☐ Autres (préciser) : ……………

Quel est votre diagnostic <u>le plus probable</u> : ………………………………

Cotez le degré de certitude sur l'échelle : 0 1 2 3 4 5 6 7 8 9 10

APRES LES RESULTATS DES BIOMARQUEURS

Date : _ _ /_ _ / _ _ _ _

4. Quel est le résultat des biomarqueurs du LCR pour la demande actuelle ?

☐ En faveur d'une MA ☐ Non en faveur d'une MA ☐ Non contributive

Aβ-42 : …………… pg/mL **Tau :** ………………… pg/mL **P-Tau :** ………………… pg/mL

5. Quelle est votre (vos) hypothèse(s) diagnostique(s) après réception des résulats de biomarqueurs du LCR ?[3]

☐ Maladie d'Alzheimer ☐ MCI[5] :……………………….. ☐ Démence fronto-temporale
☐ Démence vasculaire ☐ Démence sémantique ☐ Aphasie progressive (APL/APNF[4])
☐ Démence parkinsonienne ☐ Cause toxique (alcool) ☐ Maladie à prions
☐ Dégénérescence cortico-basale ☐ Paralysie supranucléaire ☐ Démence à corps de Lewy
☐ Troubles psychiatriques ☐ Atrophie focale progressive ☐ Autres (préciser) : ……………

Quel est votre diagnostic <u>le plus probable</u> : ………………………………

Cotez le degré de certitude sur l'échelle : 0 1 2 3 4 5 6 7 8 9 10

6. Les biomarqueurs ont-ils modifié votre diagnostic final ? ☐ Oui ☐ Non

7. Les résultats des biomarqueurs ont-ils modifié votre traitement médicamenteux ?

☐ Pas de modification
☐ Introduction de traitement type inhibiteurs de l'acétylcholine-estérase, anti-NMDA
☐ Arrêt de traitement type inhibiteurs de l'acétylcholine-estérase, anti-NMDA
☐ Autres (préciser) : ……………………………………………………………………………………

8. Les résultats des biomarqueurs ont-ils modifié votre prise en charge médico-sociale ?

☐ Pas de modification ☐ Demande d'APA ☐ Demande ALD
☐ Participation à une étude ☐ Autres (préciser) :………………………………………………

1. Mild Cognitive Impairment 2. Essais thérapeutiques, PHRC… 3. cochez <u>une ou plusieurs cases</u> ; en cas de suspicion de démence mixte, détaillez dans autres
4. Aphasie progressive primaire logopénique / non fluente 5. préciser si MA prodromale ou MCI non MA.

MERCI DE RENVOYER CE QUESTIONNAIRE PAR L'UN DES MODES SUIVANTS :

FAX au | **01 49 95 84 95** mail à | **biomarqueurlcr@gmail.com** courrier au | **CM2R Paris Nord Ile-de-France**
200, rue du Faubourg Saint-Denis 75010 Paris
